# The East Texas Medico-Chirurgical Association

**Published:** 1902-09

**Authors:** E. E. Guinn

**Affiliations:** Secretary


### The East Texas Medico-Chirurgical Association.

At the recent meeting of this society, at Palestine, the following officers were elected for the ensuing year: Dr. O. L. Hathcock, of Palestine, President; Dr. E. B. Parsons, of Palestine, First Vice-President; Dr. J. H. Joyce, Second Vice-President; Dr. J. M. Colly, Treasurer; and E. E. Guinn, Secretary.

Committee of Arrangements for November Meeting.—Dr. B. F. Moore, Chairman; B. F. Chambers and W. G. Jameson.

Chairmen of Sections.—General Medicine, Dr. W. E. Link; Obstetrics and Gynecology, Dr. E. E. Bryson; Diseases of Children, Dr. J. H. Evans; General Surgery, Dr. F. L. Barnes.

There were a number of good papers read, which will appear in the "Red-Back" from time to time. Several new members were added, and a good time had.

E. E. GUINN,
Secretary.

